# Variation Between Three *Eragrostis tef* Accessions in Defense Responses to *Rhopalosiphum padi* Aphid Infestation

**DOI:** 10.3389/fpls.2020.598483

**Published:** 2020-12-08

**Authors:** Nathan M. Gyan, Beery Yaakov, Nati Weinblum, Anuradha Singh, Alon Cna’ani, Shiran Ben-Zeev, Yehoshua Saranga, Vered Tzin

**Affiliations:** ^1^The Albert Katz International School for Desert Studies, Jacob Blaustein Institutes for Desert Research, Ben-Gurion University of the Negev, Sede Boqer, Israel; ^2^French Associates Institute for Agriculture and Biotechnology of Drylands, Jacob Blaustein Institutes for Desert Research, Ben-Gurion University of the Negev, Sede Boqer, Israel; ^3^Jacob Blaustein Center for Scientific Cooperation, Jacob Blaustein Institutes for Desert Research, Ben-Gurion University of the Negev, Sede Boqer, Israel; ^4^The Robert H. Smith Faculty of Agriculture, Food & Environment, The Hebrew University of Jerusalem, Rehovot, Israel

**Keywords:** cereal crop, electrical penetration graph, green leaf volatile, insect behavior, trichome, volatile organic compounds, aphid

## Abstract

Tef (*Eragrostis tef*), a staple crop that originated in the Horn of Africa, has been introduced to multiple countries over the last several decades. Crop cultivation in new geographic regions raises questions regarding the molecular basis for biotic stress responses. In this study, we aimed to classify the insect abundance on tef crop in Israel, and to elucidate its chemical and physical defense mechanisms in response to insect feeding. To discover the main pests of tef in the Mediterranean climate, we conducted an insect field survey on three selected accessions named RTC-144, RTC-405, and RTC-406, and discovered that the most abundant insect order is Hemiptera. We compared the differences in *Rhopalosiphum padi* (Hemiptera; Aphididae) aphid performance, preference, and feeding behavior between the three accessions. While the number of aphid progeny was lower on RTC-406 than on the other two, the aphid olfactory assay indicated that the aphids tended to be repelled from the RTC-144 accession. To highlight the variation in defense responses, we investigated the physical and chemical mechanisms. As a physical barrier, the density of non-granular trichomes was evaluated, in which a higher number of trichomes on the RTC-406 than on the other accessions was observed. This was negatively correlated with aphid performance. To determine chemical responses, the volatile and central metabolite profiles were measured upon aphid attack for 4 days. The volatile analysis exposed a rich and dynamic metabolic profile, and the central metabolism profile indicated that tef plants adjust their sugars and organic and amino acid levels. Overall, we found that the tef plants possess similar defense responses as other Poaceae family species, while the non-volatile deterrent compounds are yet to be characterized. A transcriptomic time-series analysis of a selected accession RTC-144 infested with aphids revealed a massive alteration of genes related to specialized metabolism that potentially synthesize non-volatile toxic compounds. This is the first report to reveal the variation in the defense mechanisms of tef plants. These findings can facilitate the discovery of insect-resistance genes leading to enhanced yield in tef and other cereal crops.

## Introduction

The world depends on many crop species to sustain the food supply. However, the commercialization of conventional agriculture has led to concentrating on only a few of these crops, which must be examined critically to ensure reliable food supply even with current population growth and climate change (Awika, 2011; Curtis and Halford, 2014). Approximately 50% of plant-based caloric intake is obtained from three primary grain sources—rice, wheat, and maize, while most traditional species are neglected and underutilized. Examples for underutilized cereals are broomcorn millet (*Panicum miliaceum* L.), canary seed (*Phalaris canariensis* L.), and tef [*Eragrostis tef* (Zuccagni) Trotter], which are monocotyledonous plants in the family of Poaceae (grasses), the same as the abovementioned staple crop (Bekkering and Tian, 2019). Most of these traditional crops offer an opportunity to improve agricultural production and maintain sustainable food security. Furthermore, these crops have a wealth of nutritional qualities and desirable traits that enhance their adaptability to climate change (Padulosi et al., 2012), and much more fundamental research is required to better understand them as a potential source of sustainable food production.

Tef is a small-seeded cereal millet. Tef is an allotetraploid cereal with a chromosome number of 20 (AB; 2n = 4x = 40), and its subgenomes are relatively small (∼300 Mb), with high gene density and low transposable element content (VanBuren et al., 2020). It originated in Ethiopia, where it is considered a staple crop, and the number one cereal produced in the country (Seyfu, 1993). Traditionally, it is grown by small-scale farmers; therefore, thousands of locally adapted accessions have been developed (Report on Area and Production Major Crops, 2012). The available genetic diversity in Ethiopia has driven breeding programs to improve existing varieties and meet market demand and consumers’ specifications (Ayalew et al., 2011; Assefa et al., 2015). The grains are commonly used for the preparation of a fermented sourdough bread known as “injera,” as well as for straw, feed, and to reinforce the walls of mud huts. Tef has more essential amino acids—including lysine, the most limiting amino acid—than barley, and wheat (Jansen et al., 1962; Yigzaw et al., 2004). It has high flour productivity, high market price, and adaptability to a wide range of environmental conditions (Reda, 2014). Recently, tef plants have been introduced to different parts of the world, including the United States, the Netherlands, and Israel (Assefa et al., 2011).

Millets such as tef face several production constraints since they are mostly cultivated in marginal areas with low moisture and limited fertility conditions (Dosad and Chawla, 2018). Inherent characteristics, such as susceptibility to pests and diseases, can cause a significant yield loss (Assefa et al., 2011; Ben-Zeev et al., 2020). One of the main reasons for crop loss is pests, which cause an average 15% reduction in grain quality and yield (Lee et al., 1981; Deutsch et al., 2018). Aphids (Hemiptera: Aphididae), of which there are approximately 5,000 species worldwide, are a dominant pest of cereal crops (Vickerman and Wratten, 1979; Rabbinge et al., 1981). This pest affects plant production through the reduction of nutrients, diminished photosynthetic efficiency, modification of sink-source ratio (Bing et al., 1991; Zhou et al., 2015), and transmission of plant viruses (Fereres et al., 1989; Nault, 1997). The aphids are phloem-feeding insects that use their stylets to penetrate the host tissues, causing minimal tissue damage (Douglas, 2003). Once an aphid finds a suitable feeding site, it can ingest phloem sap for hours or even days and adapt to the phloem sap compound composition (Nalam et al., 2020). There is limited knowledge about tef pests in general and aphids in particular. To reduce pest damage, plants have evolved defense strategies, that can be present constitutively or be induced on demand (Agrawal, 1999; Mithöfer and Boland, 2012). Some of the main strategies commonly present in the Poaceae family plant species include: (i) physical barriers, (ii) metabolic adjustments to modify the food source consumed by aphids, and (iii) chemical defenses and signals (volatiles and non-volatiles). The physical barrier on the leaf surface is the key interface between plants and insects that interrupts insect feeding. Many surface characteristics, including the trichomes, cuticle, epidermis, waxes, and cell walls, can modulate these interactions (Agrawal et al., 2009). The leaf surface of young wheat and barley plants are covered with non-glandular trichomes, specialized epidermal hair-like structures, that might affect aphid movement and reproduction rate (Leybourne et al., 2019; Batyrshina et al., 2020b; Correa et al., 2020). To cope with insect attack, plants adjust their central metabolism by transiently modifying photosynthetic efficiency and remobilizing carbon and nitrogen resources (Meihls et al., 2012; Appel et al., 2014). The Russian wheat aphid (*Diuraphis noxia*) infestation on wheat leaves has caused significant losses of chlorophyll *a* and *b* and carotenoids (Ni et al., 2002). In barley leaves, 30 genes associated with photosynthesis were inhibited after 3 h of feeding (Gutsche et al., 2009). The metabolite content in the phloem sap can be adjusted in response to aphid feeding (Leybourne et al., 2019). For example, the feeding of greenbug aphids (*Schizaphis graminum*) on wheat leaves enhances the content of essential amino acids in the phloem sap (Dorschner et al., 1987; Sandström et al., 2000).

In response to insect attack, plants adjust not only their central metabolites but also synthesize specialized deterrent metabolites that can affect the insect nervous, digestive, and endocrine systems (Eisner et al., 2000; Meihls et al., 2012; Fürstenberg-Hägg et al., 2013). In the Poaceae family, substrates from the shikimate pathway, mainly indole-, and Tyr-derived compounds, serve as a source for various classes of specialized deterrent metabolites. This includes: (i) benzoxazinoids in wheat and maize (Frey et al., 1997), (ii) gramine in cultivated barley (Grün et al., 2005), (iii) serotonin and melatonin, detected in rice, and *Echinochloa esculenta* (Japanese barnyard millet) (Ishihara et al., 2008; Lu et al., 2018), and (iv) the cyanogenic glucoside dhurrin in Sorghum (Zhu-Salzman et al., 2004). However, none of these specialized metabolites were previously reported to be synthesized in tef plants. Another chemical response is the biosynthesis and emission of volatile organic compounds (VOCs) (Dicke, 1999). VOCs are released into the atmosphere and act as long-distance cues for herbivore deterrence, natural enemy attraction, or even serve as host-finding signals for the herbivores themselves (Gershenzon and Dudareva, 2007; Bleeker et al., 2009). The VOCs are composed of a blend of metabolites from diverse chemical groups: (i) terpenoids, (ii) fatty acids (FAs) derivatives including methyl jasmonate, and green leaf volatiles (GLVs), (iii) indole- and Phe-derived phenolic products including methyl salicylate, (iv) methanol; and (v) ethylene (Kant et al., 2009). Most studies conducted on plants from the Poaceae family have suggested that the mono-, sesqui-, and di-terpenoids, and FAs are the main VOCs that are modified in response to herbivory (Richter et al., 2015, 2016; Ameye et al., 2018) as well as methyl salicylate (Stepanycheva et al., 2016).

Here, we characterized what are the pests that feed on tef in Israel, and how the plants defend themselves against these pests. Plant genotypes (accessions or lines) can widely differ in their molecular responses to aphids (Song et al., 2017). We hypothesize that tef plants evolved defense mechanisms similar to other Poaceae plant species, that can vary between tef accessions. To reveal the variety and effectiveness of tef defense mechanisms, we used three tef accessions. We started this study by elucidating the overall insect abundance on tef in the field, then focused on one pest, the bird cherry-oat aphid (*Rhopalosiphum padi* L.), which is among the most agriculturally devastating aphids worldwide (Blackman and Eastop, 2000; Parry, 2013). We analyzed the differences in insect performance and preference, trichome density, and metabolic and transcriptomic changes in response to aphid attack. We discovered that tef plants rely on both physical and chemical defenses and adjust their central metabolism in repose to aphid attack. Our work is the first report to highlight the defense mechanisms of tef plants in response to herbivore attack on the molecular level. These findings could be further utilized to reduce pesticide applications and breed accessions with enhanced resistance.

## Materials and Methods

### Plant Material, Field Experiment, and Insect Survey

Three tef accessions, RTC-144, RTC-405, and RTC-406, were selected from the available germplasm (Ben-Zeev et al., 2018). Among 273 tef accessions examined in this field study, both RTC-405 and RTC-406 were found suitable for Mediterranean climate and used as standards in our earlier trials. RTC-144, which is also named “Magna,” is an improved variety that was previously used as a part of 20 tef cultivars panel, for discovering novel, simple sequence repeat (SSR) markers (Cannarozzi et al., 2014). The plant phenotypes, and seed color of the three accessions are presented in Supplementary Figure S1. Field experiments were conducted at two research sites: (i) Sede Boqer campus, southern Israel (30.87417°N, 34.79639°E), and (ii) Revadim, central Israel (31.772576°N, 34.806949°E). The Sede Boqer experiment consisted of three 1 m^2^ plots of each of the three tef accession, randomly positioned with 1 m distance between plots. Water was provided once a week, either via rainfall or irrigation. Fertilizer was provided as previously described (Batyrshina et al., 2020a), and no pesticides or herbicides were applied during the experiments. The Revadim experimental site included a total of 21 accessions sown in a randomized block design with four replicates. Each plot was 8 m long by 1.93 m wide. Water was applied once a week using a sprinkler irrigation system. All management operations (soil preparation, irrigation, and pesticide application), were conducted according to the commercial growing protocol adopted by local farmers in Israel. Only two accessions were grown in this site, RTC-405 and RTC-406. The insect survey was conducted by holding the Vortis^TM^ suction sampler (Burkard Manufacturing Co., Ltd., United Kingdom) above the plants across the 1 m^2^ plot (Sede Boqer), and along 15.4 m^2^ (Revadim) and vacuuming at maximum suction power for 30 s into a 50 mL collection tube (Arnold, 1994; Zentane et al., 2016). Sampling was done prior to flowering (late May 2019), and during flowering (late June 2019). Insects were subsequently kept in 2–3 mL of 70% ethanol, transferred to 9 cm diameter Petri dishes, then observed by stereomicroscope (Nikon SMZ745, Nikon Instruments Inc., United States) under 10x magnification. The insects were sorted by order level, using the Key to Insects Orders (extension.colostate.edu/Gardennotes/315.pdf) and family level (Hamilton et al., 2012; Zettler et al., 2016), and normalized for insect order per square meter of tef plants (Supplementary Table S1 and Supplementary Figure S2).

### Plant Growth in Laboratory Conditions

Several dozen tef seeds were sown on moistened soil mix [tuff mixture with vermiculite (2:1) and an N-P-K fertilizer] in 330 cm^3^ plastic pots, maintained under controlled growth conditions with a light regime of 12 h light/12 h dark photoperiod at a constant room temperature of 26–28°C, relative humidity of 60–70%, and an average light intensity of 300 μmol photons m^–2^ s^–1^. After 2 weeks, the seedlings were transplanted into individual plastic pots, and the same growth conditions were maintained.

### Aphid Non-choice Bioassay

The bird cherry-oat aphids (*Rhopalosiphum padi*) were collected from the field in Spring 2017, and the colony was reared on tef plants (accession RTC-144) under controlled conditions, as mentioned above. For the aphid reproduction bioassay, 20 adult aphids were applied onto 1-month-old tef plants for 4 and 7 days (14–15 biological replicates were tested at each time point and accession). The total number of aphids was counted (total nymphs and adults) and divided by the initial number of adults. The bioassays were conducted in a whole cage bioassay where plants were covered with plastic bags (Cryovac Crispac Beutel Super Micro Lochung 15 × 60 cm; Baumann Saatzuchtbedarf, Germany). After infestation time, tissue samples were harvested and flash frozen in liquid nitrogen, then stored at −80°C for further metabolic analysis.

### Aphid Choice Bioassay Using a Y-Shape Tube Olfactometer

The Y-shape olfactometer was built as previously described (Akol et al., 2003), with several adjustments. It was comprised of a 21 cm-long base with an internal diameter of 3.5 cm and two lateral 15 cm branches at an angle of 75°connected to a 10 L glass beaker in which the odor source was held (see Supplementary Figure S3). The tef plants were held for 1 h in the glass beaker as a source of volatiles, and air was provided at 0.8 L min^–1^ to both branches of the Y-tube via an air pump. One adult aphid was released within the base of the Y-tube with a paintbrush after being starved for 2 h. The aphid choice was conducted up to 5 min, and an aphid that walked halfway or more toward the Y-tube lateral branches was reported as a responsive individual. A 20W fluorescent light was placed 0.5 m above the Y-tube olfactometer in a controlled environment (25°C and 60% relative humidity) to disable the insect’s vision. The positions of the volatile sources were alternated between replicates to eliminate directional bias. All glassware and Y-tubes were cleaned and sterilized with 70% ethanol before new plants were used to reduce the risk of contamination by previously tested volatiles. Overall, the test was repeated five times for each pair of odor sources, with 30 adult aphids. As a control, aphids were introduced to the same accession (RTC-405) from both sides of the Y-tube, which had shown no significant differences, indicating that the olfactometer system is balanced.

### Aphid Feeding Behavior Recorded by the Electrical Penetration Graph (EPG) System

Aphid feeding behavior was monitored on two tef accessions, RTC-144 and RTC-406, using the EPG on a Giga-8dd system (Wageningen, Netherlands). A gold wire (18 μm diameter) was attached to the dorsal surface of each *R. padi* aphid’s abdomen using silver glue (Salvador-Recatalà and Tjallingii, 2015). One-month-old tef plants were placed into a Faraday cage, electrodes were placed into the soil, and the insect probes were adjusted, allowing for contact between the leaf surface and the insect. Voltage waveforms were digitized at 100 Hz with an A/D converter, and patterns were identified as previously described (Tjallingii, 1978; Tjallingii and Esch Hogen, 1993). Waveform recordings were dissected every 30 s with the EPG analysis software StyletD installed in a computer connected to a Giga direct current amplifier. The parameters measured were comparable to those categorized by Sarria et al. (2009): (i) time until first probing (t_1Pr), (ii) xylem–including duration (s_G), and number of occurrences (n_G); (iii) phloem–including the total duration of E1 followed by E2 (s_E1– > E2), the total duration of E (s_E), number of E1 occurrences (n_E1), and number of E2 occurrences (n_E2); (iv) all tissue–including the total duration of C occurrences (s_C), the total duration of non-probing occurrences (s_NP), the total duration of potential drops occurrences (s_PD), number of probing occurrences (n_Pr), number of non-probing occurrences (n_NP), and number of potential drop occurrences (n_PD). The pathway phase analyzed A, B, and C were not calculated separately. EPG waveforms and results were analyzed using StyletA software as previously described (Nalam et al., 2018), and Excel for automatic parameter workbook calculation (Sarria et al., 2009). The data for the four phases was recorded for 6 h, while after the 4–5 h, plant rejections were observed. Therefore, we analyzed the first 3 h, where the significant possible sequence of feeding differences was detected (Marchetti et al., 2009). Overall, 15 plants from each accession were tested.

### Determination of Trichome Density on Leaf Surfaces

Tef plants from the three accessions were grown for 1 month (no aphids were applied on these leaves). Then, 2 cm sections were sampled from the widest part of three leaves: (i) lower leaf (a first leaf from the base), (ii) middle leaf, and (iii) upper leaf. The three leaves were dissected, bleached in 80% (v/v) ethanol, boiled at 90°C for 20 min, and washed with distilled water as previously described (Batyrshina et al., 2020b). For trichome visualization, leaves were mounted on microscope slides with the adaxial side facing up, covered with glass coverslips. A digital camera connected to an Axioplan 2 Upright Light Microscope (Zeiss, Oberkochen, Germany) was used for imaging. For each tef accession, five biological replicates with two pictures per leaf were taken. For density quantification, trichomes were counted using ImageJ software^1^ and normalized per mm^2^.

### Volatile Organic Compound (VOC) Analysis

One-month-old plants of the three accessions were infested with *R. padi* aphids for 4 days, and tissue samples were harvested and immediately frozen in liquid nitrogen and stored at −80^*o*^C. Then, 1 g of frozen tissue was ground and added to a 20 mL glass vial (Chrom4, Thüringen, Germany), containing 0.8 μg isobutylbenzene internal standard (Sigma-Aldrich, Israel), 7 mL NaCl (20%), and 1 g NaCl. A divinylbenzene/carboxen/polydimethylsiloxane (DVB/CAR/PDMS 50/30 μm, Supelco/Sigma-Aldrich, Israel) solid-phase micro-extraction (SPME) fiber was used to collect VOCs. Since tef volatiles have not been previously studied, a broad survey to reveal all potential VOCs was selected. C2–C20 n-alkane size standards were added to the samples (Garcia-Esteban et al., 2004). A COMBI PAL-XT (CTC Analytics AG, Switzerland) auto-sampler/robot for Agilent gas chromatography (GC) 7890 connect to mass spectrometry (MS) 5977b was used. Glass vials were heated at 60°C for 15 min prior to sampling, after which the fiber was inserted into the vial headspace for an additional 15 min at the same temperature. The vial needle penetration was 11 mm. The injection volume was 10 μL, the needle penetration was 32 mm, and the injection fiber exposure was 22 mm for an absorption time of 10 min. The analytes were then desorbed by heating the fiber in the injection port of a GC-MS to 250°C for 3 min. The analytes were separated on a VF-5MS + 10 m EZ guard capillary column (30 m × 0.25 mm × 0.25 μm; Agilent CP9013, United States). The oven temperature program was as follows: 40°C initially for 1 min, increased to 250°C at 6°C/min, followed by a post-run 280°C for 5 min. Helium was used as a carrier gas at a constant flow rate of 1 mL min^–1^. Injection temperature was set to 270°C (splitless mode), the transfer line temperature was 280°C and the ion source was adjusted to 230°C. Mass spectra were collected at 2.1 scans s^–1^ with a scanning range of 40–400 mass−to−charge (m/z) ratio and electron energy of 70 eV. Extracted compounds were tentatively identified based on Wiley 10 with NIST 2014 mass spectral library data using the MassHunter software package (version B.10.0.368, Agilent, United States). Further compound identification was based on a comparison of mass spectra and retention times with authentic standards (Sigma−Aldrich, Israel) analyzed under similar conditions. Compounds that could not be identified using standards were designated as “Unidentified,” followed by their putative class (Supplementary Tables S2, S3). For each tef accession, 4–5 biological replicates were analyzed.

### Central Metabolite Analysis

One-month-old plants of the three accessions were infested with *R. padi* aphids, or kept uninfested as control, following the non-choice whole cage bioassay as described above. After 4 days, the samples were harvested and immediately flash frozen in liquid nitrogen, and stored at −80°C. Then metabolites were extracted using 100 mg of ground frozen plant tissue mixed with a methanol/water/chloroform solvent at a ratio of 55:23:22 (v/v/v) following a previously described protocol, with minor modifications (Rosental et al., 2016). In brief, the top 300 μL of hydrophilic layer was collected and dried in a vacuum. For derivatization, 40 μL of 20 mg/mL methoxyamine hydrochloride (Sigma-Aldrich, Israel) was added, dissolved in pyridine, and incubated for 2 h in an orbital shaker at 37°C. Next, N-methyl-N-(trimethylsilyl) tri-fluoroacetamide (MSTFA), including an alkane standard mix in a volume of 77 μL, was added to each sample, followed by a 30 min incubation in an orbital shaker at 37°C. Finally, 1 μL of the sample was injected into the Agilent 5977B GC-MS instrument. Data acquisition was conducted using the Mass Hunter software, NIST mass spectral library, and retention index (RI) libraries^2^ (Lisec et al., 2006; Hochberg et al., 2013). Each metabolite was normalized to D-sorbitol (^13^C_6_) as an internal standard and presented as the relative abundance of the ion counts (Supplementary Tables S4, S5). For each tef accession, 4–5 biological replicates were analyzed.

### RNA Extraction, Transcriptome Sequencing and Analysis

One-month-old RTC-144 tef plants were infested with 20 adult *R. padi* aphids for 6, 24, and 96 h as well as uninfested control using a non-choice whole cage bioassay as described above. All plants were caged at the beginning of the experiment, and the addition of aphids was staggered so that the leaf tissues for gene expression were harvested at the same time (96 h after the start of the experiment). For each time point, three replicates were generated. Total RNA was extracted using an SV Total RNA Isolation Kit with on-column DNaseI treatment (QIAGEN), then purified and quantified. For next-generation sequencing, 2.5μg of each sample was used. The paired-end (150 bp read length) RNAseq was conducted using an Illumina HiSeq 4000 instrument by GeneWIZ Inc.^3^ Quality control was performed using FASTQC. Adapters and low-quality sequences were trimmed and excluded using Trimmomatic v0.36. Then, mapping was performed using STAR aligner v2.5.2b against the *Eragrostis tef* reference transcriptome version 1.0 (Cannarozzi et al., 2014). Reads aligning to exons were retrieved using Subread v1.5.2. Differential gene analysis was performed using DESeq2 v1.22.2 (Love et al., 2014), via a likelihood ratio test to evaluate multiple genotypes at once (adjusted *p* < 0.05). The data was transformed using rlog (Supplementary Table S6). GO annotations were extracted by comparison with the SwissProt annotation of tef genes provided by Cannarozzi et al. (2014) to functional annotation of SwissProt entries. Gene expression fold change was calculated by dividing each value by the average of the gene control samples. The raw sequence data have been submitted to NCBI Sequence Read Archive (SRA) accession PRJNA623870.

### Statistical Analysis

The olfactometer results were examined by chi-square goodness of fit test at *p* < 0.05. The EPG parameters were compared between the two accessions using a paired Student’s *t*-test, *p* < 0.05. Differences in aphid reproduction using a non-choice bioassay and trichome density among accessions at each time or leaf section, were analyzed by two-way ANOVA (analysis of variance), and one-way ANOVA (each time point or leaf section, respectively), followed by a *post hoc* test using TukeyHSD, corrected with the false discovery method. These analyses were conducted by JMP13 software (SAS)^4^, and figure presentations were done in Microsoft Excel. For the VOC and central metabolic analysis, the raw data were normalized using the MetaboAnalyst software using the following steps: observations missing more than 50% of value estimation features were removed and replaced by a small value that was calculated as half of the minimum value of the original data, and the interquartile range data was filtered, then normalized to the median, transformed into log scale, and auto-scaled (Xia et al., 2009). The normalized data was used for the heatmap, the two-way ANOVA, and the paired Student’s *t*-test (*p* < 0.05) corrected with the false discovery method. These analyses were conducted by MetaboAnalyst. The principal component analysis (PCA) and Venn diagrams were calculated and designed using R. For the heatmap, the Euclidean distance with Ward’s minimum variance method was calculated using the default parameters.

## Results

### Insect Abundant on Three Selected Tef Accessions in the Field

**FIGURE 1:**
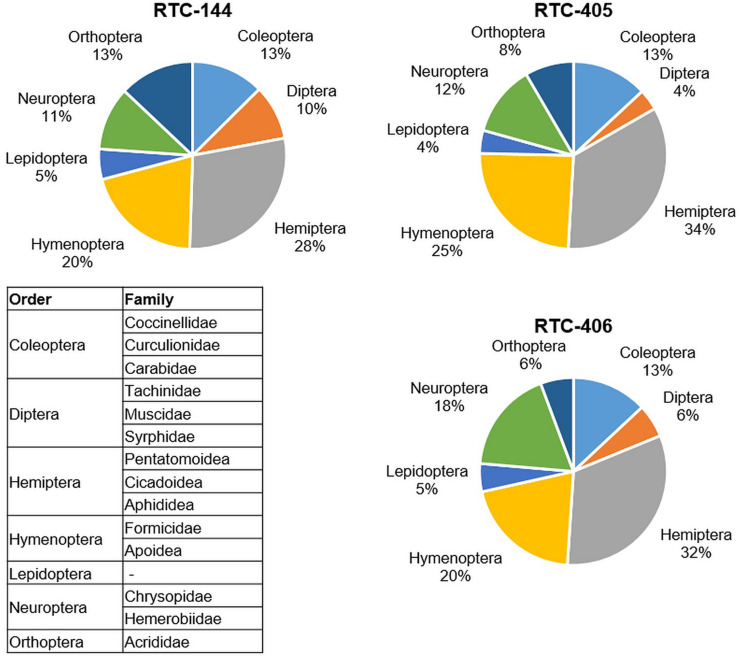
Insect abundance on the three tef accessions from the field survey. Pie chart of the insects that were collected in three locations in the field (total of 3 m^2^ and normalized to 1 m^2^) sorted by orders. The insect families monitored in each order are included in the table. Sampling was performed prior to flowering (late May 2019).

In the first sampling date (May 2019; prior to tef flowering) at the Sede Boqer field insect survey, seven insect orders were detected on tef plants: Coleoptera, Diptera, Hemiptera, Hymenoptera, Lepidoptera, Neuroptera, and Orthoptera (Figure 1). The largest number of insects counted on all three tef accessions were of Hemiptera, including three families: Pentatomoidea, Cicadoidea, and Aphididea, the smallest number belonged to Lepidoptera. The survey indicated differences in insect abundance between the three tef accessions, wherein Orthoptera showed more than twofold differences between the tef accessions (13% to RTC-144 relative to 6% to RTC-406 from the total insects per accession). The Dipteran order, including the superfamily Tachinidea, and families Muscidae and Syrphidae, play an essential role in various trophic levels both as pests of crops, as well as pollinators (Rader et al., 2016). The Dipteran order showed the most diversity between the three tef accessions and was 2.5 times more abundant on RTC-144 (10%) than RTC-405 (4%). A similar trend was detected on the second sampling date (June 2019) in Sede Boqer, as well as in the Revadim site (Supplementary Table S1). Results of both sampling dates and sites emphasize that the insects from the Hemiptera order are highly abundant on tef plants in this geographic region, 23–34% in Sede Boqer, and 30–38% in Revadim from the total insects per accession.

### The Difference in Aphid Preference, Performance, and Feeding Behavior on Tef Leaves Under Controlled Growth Conditions

**FIGURE 2:**
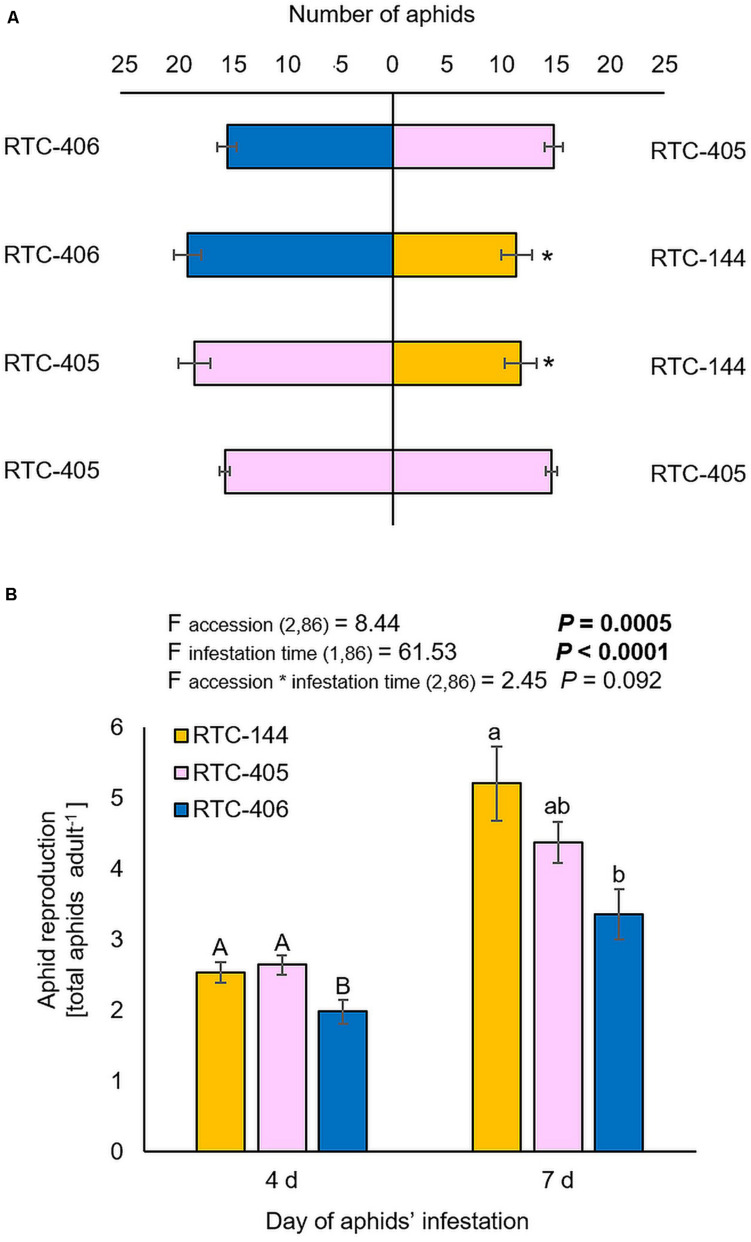
Aphid performance and preference for 1-month-old tef plants. **(A)** A Y-tube olfactometer choice bioassay was used to determine the aphid preference. Bars represent the average number of aphids (mean ± SE, *n* = 5). In each replicate, 30 aphids were tested. The asterisk indicates significantly different choices as determined by the chi-square goodness of fit test at *P* < 0.05. **(B)** A non-choice bioassay was used to determine the differences in aphid performance between the three tef accessions. The whole-plants were infested with 20 adult *R. padi* aphids for 4 and 7 days, then the total number of adult and nymphs was counted (mean ± SE, *n* = 14–15). On the top, a summary of the two-way ANOVA, comparing the aphid reproduction among the three accessions at two infestation time periods 4 and 7 days (*p* < 0.05). Different letters above the bars indicate significant differences, using one-way ANOVA followed by TukeyHSD test separately for each time point, corrected with the false discovery method.

The bird cherry-oat aphid (Hemiptera; Aphididae; *Rhopalosiphum padi*), is highly abundant on host plants from the Poaceae family (Swirski and Amitai, 1999). Thus, we characterized tef defense responses by focusing our laboratory experiments on a single aphid species, *R. padi.* First, we performed a choice bioassay using a Y-shape olfactometer. The results showed that aphids tended to be repelled by accession RTC-144 compared to either RTC-405 or RTC-406, while no preference between the two later accessions, RTC-405 and RTC-406 were observed (Figure 2A). Additionally, we evaluated the aphid reproduction on the three tef accessions at two infestation time points, 4 and 7 days, using a non-choice bioassay (Figure 2B). The two-way ANOVA suggested a significant difference between the three tef accessions (F_*accession* 2_,_86_ = 8.44, *p* = 0.0005), the time of aphid-infestation (F_*time* 1_,_86_ = 61.53, *p* < 0.0001), but no significant interaction between the two factors (F_accession__*__time 2_,_86_ = 2.45, *p* = 0.092). A one-way ANOVA of the aphid number at each time point indicated that after a 4 days infestation, the number of aphids was significantly lower in the RTC-406 accession relative to the other two accessions, while after 7 days, there was only a significant difference between RTC-144 and RTC-406.

Lastly, we investigated aphid feeding behavior using the electrical penetration graph (EPG) technique (Tjallingii and Esch Hogen, 1993). We conducted this experiment on two selected accessions, RTC-144 and RTC-406, which possessed opposite trends in performance and preference (Figure 2). Parameters from the four phases were recorded, including epidermis, xylem, phloem, and all tissues (the phases were categorized by Sarria et al., 2009). As shown in Table 1, three variables were significantly different between the tef accessions. The time to first probe from the start of EPG (t_1Pr) was significantly longer in RTC-406 (13.05 min) than RTC-144 (4.59 min). The number of xylem events (n_G) was larger on RTC-144 (2.58 times) than RTC-406 (1.29 times), and the total duration of non-probing (s_NP) was longer for RTC-406 (22.30 min) than RTC-144 (11.82 min). Altogether, the results indicated that the variation in aphid performance and feeding behavior between the tef accessions might be due to multiple factor defense responses. Thus, we performed several experiments to reveal these factors, including evaluating the physical barriers related to the time to first probing, and central and specialized metabolites that might affect reproduction. Additionally, we quantified volatile content, as their potential emission can affect aphid preference from a long distance.

**TABLE 1:** Feeding behavior of *Rhopalosiphum padi* on two tef accessions using the electrical penetration graph (EPG) technique.

Phase	Parameter full name	Parameter short name	Unit	RTC-144 (mean ± SE)	RTC-406 (mean ± SE)	*p* value
-	**Time to 1st probe from start of EPG**	t_1Pr	min	4.59 ± 2.14	13.05 ± 2.12	**8.89E-03**
Xylem	Duration of G	s_G	min	31.81 ± 4.89	23.35 ± 6.61	3.27E-01
	**Number of G**	n_G	#	2.58 ± 0.45	1.29 ± 0.16	**8.50E-03**
Phloem	Total duration of E1 followed by E2	s_E1– > E2	min	3.19 ± 0.78	6.13 ± 1.28	6.47E-02
	Total duration of E	s_E	min	29.63 ± 3.98	30.06 ± 6.51	9.56E-01
	Number of E1	n_E1	#	6.33 ± 1.27	6.60 ± 0.83	8.61E-01
	Number of E2	n_E2	#	2.93 ± 0.59	3.53 ± 0.39	4.03E-01
All tissue	Total duration of C	s_C	min	109.75 ± 4.83	105.22 ± 7.08	6.01E-01
	**Total duration of non-probing**	s_NP	min	11.82 ± 4.02	22.30 ± 3.09	**4.82E-02**
	Total duration of potential drops	s_PD	min	1.56 ± 0.60	0.63 ± 0.06	1.09E-01
	Number of probes	n_Pr	#	4.00 ± 0.48	4.33 ± 0.54	6.48E-01
	Number of non-probing	n_NP	#	4.00 ± 0.48	4.33 ± 0.54	6.48E-01
	Number of potential drops	n_PD	#	14.00 ± 1.95	11.13 ± 0.94	1.79E-01

**Waveforms were analyzed using StyletA software, and Excel for automatic parameter workbook for calculation (Sarria et al., 2009). In bold are significantly different parameters (Student’s t-test, p < 0.05). Overall, 15 biological replicates from each tef accession were tested.**

### Non-granular Trichome Density on the Leaf Surface as a Physical Barrier Factor

**FIGURE 3:**
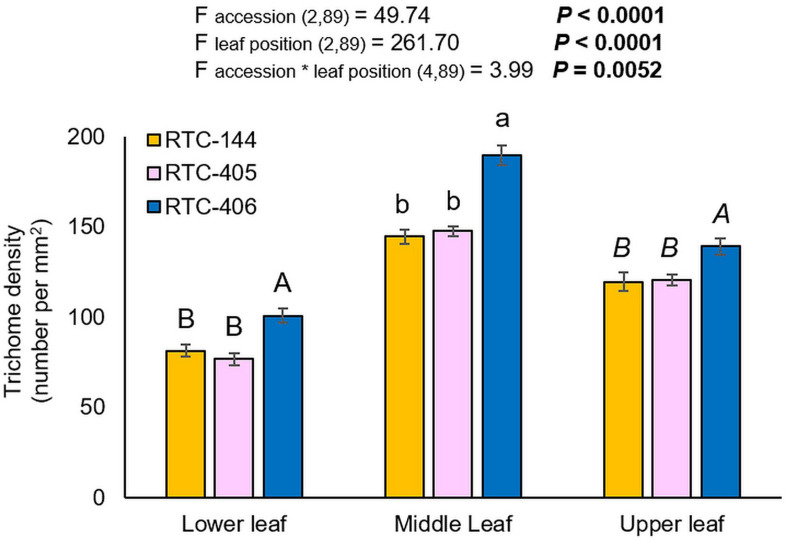
Trichome density of three tef leaves. Bars represent the average number of trichome density per mm^2^ (mean ± SE, *n* = 10). On the top, a summary of the two-way ANOVA, comparing the number of trichome among the three accessions at three leaf sections (*p* < 0.05). Different letters above the bars indicate significant differences, using one-way ANOVA followed by TukeyHSD test separately for each time point, corrected with the false discovery method.

The trichome density was evaluated on the lower, middle, and upper leaves of the main tiller. The experiment was conducted on uninfested leaves, and therefore, represent the constitutive trichome levels. As presented in Figure 3, the two-way ANOVA suggested a significant difference in trichome number between tef accessions (F _*accession* (2_,_89__)_ = 49.74, *p* < 0.0001), leaf position (F _*leaf position* (2_,_89__)_ = 261.70, *p* < 0.0001), and a significant interaction between the two factors (F _accession*__leaf position (4_,_89__)_ = 3.99, *p* = 0.0052). Between three tef accessions, the number of trichomes was significantly higher in the middle leaf than on the lower and upper leaf. Next, we analyzed the differences in trichome density at each leaf position between accessions, using one-way ANOVA. The results revealed that RTC-406 possessed the highest number of trichomes on all three leaves compared to the other two accessions. The high trichome density of RTC-406 can limit aphid feeding and cause a reduction in their reproduction.

### Constitutive and Inducible Metabolic Levels of Tef Leaves Under Aphid Attack

**FIGURE 4:**
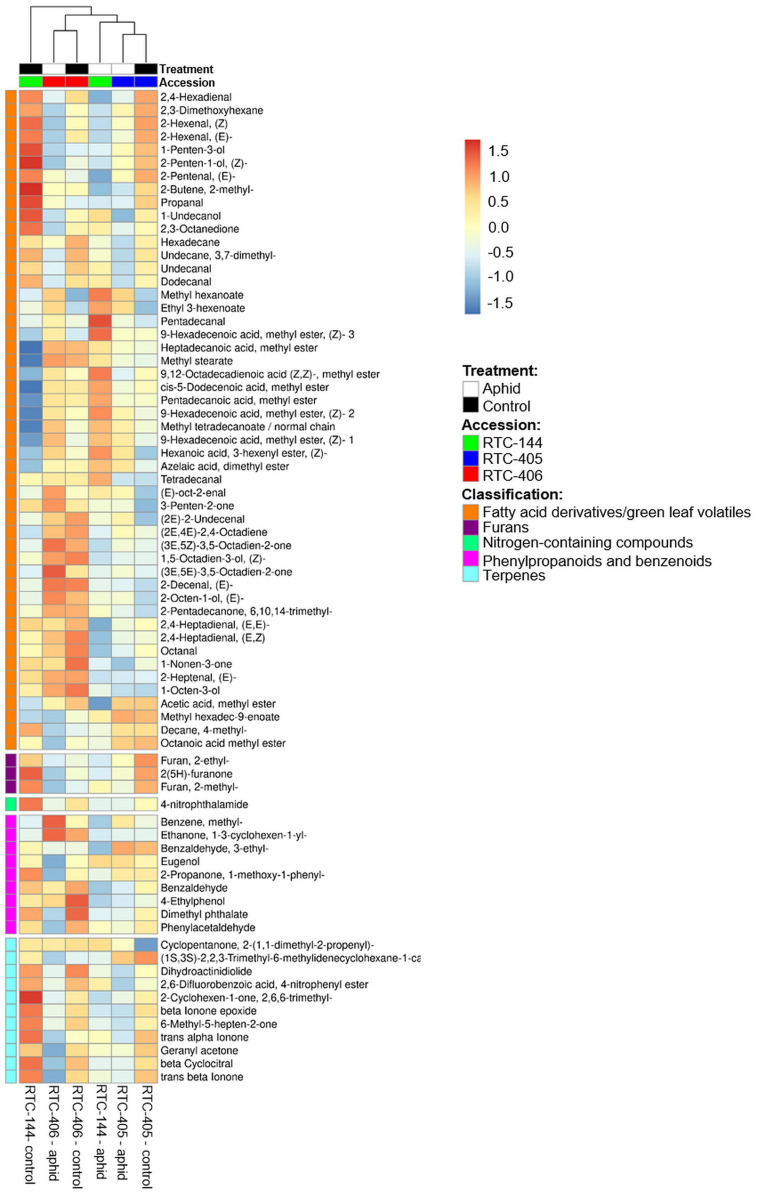
Heatmap of the VOC profile of aphid-infested and untreated control tef plants. The VOCs were selected using two-way ANOVA comparing the three accessions and the aphid treatment. The Euclidean distance with Ward’s minimum variance method was calculated using the default parameters of the MetaboAnalyst software, and the graph was created in R and presented in average values. Colors correspond with concentration values (autoscaled parameters), where red indicates high levels, and blue indicates low levels (*n* = 4–5 biological replicates).

The olfactometer experiment indicated that aphids respond according to the variation in tef’s volatile organic compound (VOC) profile, which conveys long-distance signals. Thus, we analyzed the tef VOC profiles of aphid infested plants using solid-phase micro-extraction (SPME) coupled with GC-MS. In total, 105 VOCs were identified and classified into five main chemical groups: fatty acid (FA) derivatives (including green leaf volatiles; GLVs), furans, terpenoids (mono-, and irregular terpenes), phenylpropanoids and benzenoids, and an unidentified nitrogen-containing compound. A two-way ANOVA analysis revealed 74 metabolites that were different in one of the factors: accession and aphid treatment, or an interaction between the two factors (Supplementary Table S3). A heatmap of the normalized value of these 74 metabolites is presented in Figure 4. The results revealed that treated and untreated RTC-405 and RTC-406 accessions were clustered together, while the aphid-treated and untreated RTC-144 samples were grouped separately. In the RTC-144 accession, almost half of the VOCs, belonging to classes of FA derivatives (aldehydes, ketones, and alcohols), terpenes, and furans, decreased under aphid attack, while the ester FA derivatives increased. The VOC changes were slight in RTC-405 and RTC-406 accessions. To detect the changes induced in response to aphids, paired *t*-tests were conducted between aphid-treated samples relative to untreated control in each accession separately. Table 2 presents only metabolites with at least twofold changes, and *p* < 0.05, false discovery rate (FDR) adjusted. RTC-144 showed a massive modification in the VOC levels, including a reduction in aldehyde-, ketone-, and alcohol FA derivatives, furans and phenylpropanoid and benzenoid classes, and induction in the ester FA derivatives. In RTC-405, only three metabolite levels were altered, including 2-methyl-2-butene and ethyl 3-hexanoate and methyl hexanoate, which, together with methyl hexanoate, were the only metabolites significantly increased in all three accessions. Altogether, this suggested that tef leaves possess a rich and unique blend of VOCs, which was largely modified in response to aphid infestation, especially in the RTC-144 accession.

**TABLE 2:** Volatile organic compounds significantly modified in response to aphid feeding in at least one tef accession.

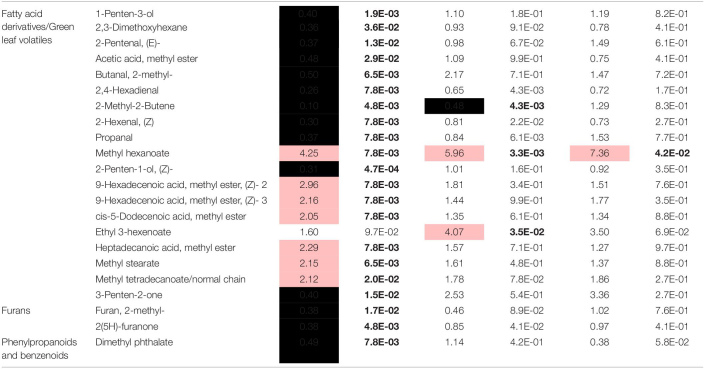

**FC, fold change > 2 (red) and < 0.5 (blue). Student’s t-test was conducted on normalized data (median and log value, as described in “Materials and Methods” section), *p* < 0.05, false discovery rate marked in bold (n = 4–5).**

**FIGURE 5:**
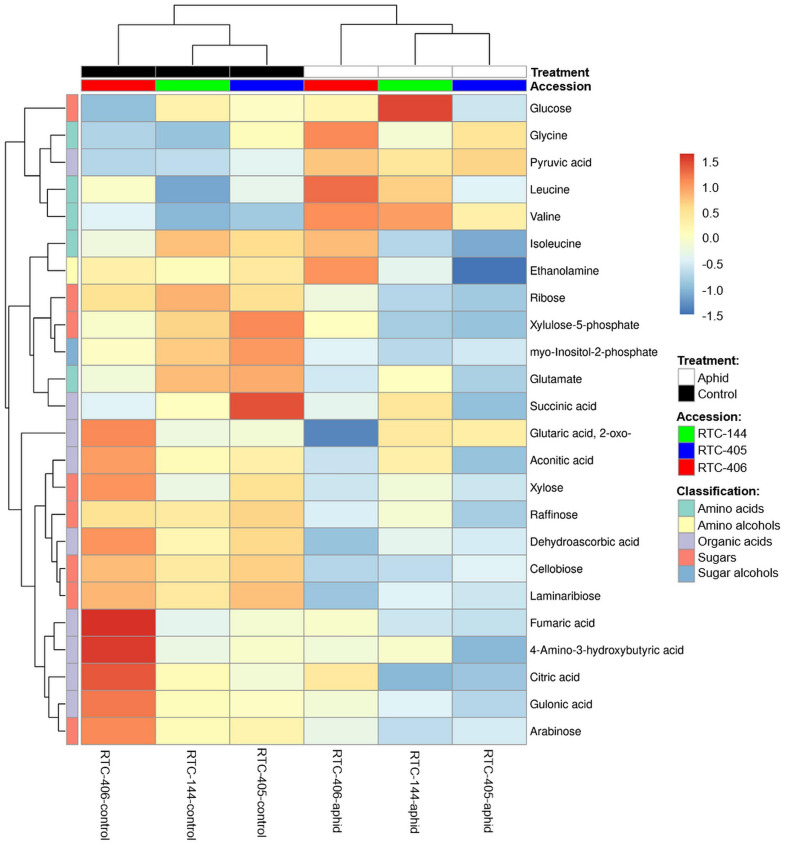
Heatmap of the central metabolites profile of aphid-infested and untreated control tef plants. The Euclidean distance with Ward’s minimum variance method was calculated using the default parameters of the MetaboAnalyst software, and the graph created in R. Colors correspond with concentration values (autoscale parameters), where red indicates high levels and blue indicates low levels (*n* = 4–5 biological replicates).

We also characterized the central metabolite profiles of the three tef accessions and their adjustment to aphid feeding after 4 days of infestation, using GC-MS. The levels of 65 metabolites were detected, including amino acids, amino alcohols, lipids, nucleotides, organic acids, sugars, and sugar alcohols (Supplementary Table S4). A two-way ANOVA analysis revealed a total of 24 metabolites that were either significantly different between the accessions, in response to aphid infestation, and/or interaction between the two factors (Supplementary Table S4). Figure 5 presents a heatmap of the average value of these 24 metabolites. The levels of most of the sugars and organic acids, as well as glutamate and myo-inositol-2-phosphate, were high in the untreated plants. Upon aphid feeding, the levels of most of the sugars, organic acids, and the amino acid Gln declined, while the levels of most of the amino acids (Gly, Leu, and Val), and the organic acid pyruvate increased. This trend was strongest for accession RTC-406. To determine the inducible effect of aphid infestation, paired *t-*tests were performed, and FDR adjusted (*p* < 0.05) on metabolites with at least a twofold change. As presented in Table 3, the RTC-405 accession showed a significant reduction in organic acid, succinic acid, and two sugars (raffinose and xylulose-5-phosphate), while only Val was significantly elevated in RTC-144. RTC-406 showed a significant reduction in cellobiose, laminaribiose, and 2-oxoglutaric acid, while glucose and Val were increased. Altogether, this suggested that the composition of the central metabolites in the tef plants slightly shift from carbon-rich compounds such as sugars and organic acids, toward nitrogen-containing compounds such as amino acids, upon aphid feeding. The metabolic changes are more pronounced in RTC-406 than RTC-144 and RTC-405.

**TABLE 3:** Central metabolites significantly modified in response to aphid feeding in at least one tef accession.

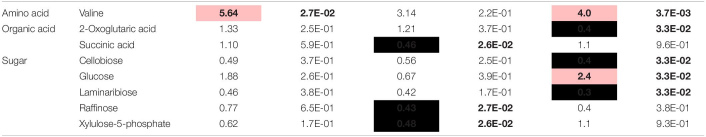

**FC, fold change > 2 (red) and < 0.5 (blue). Student’s t-test was conducted on normalized data (median and log value as described in “Materials and Methods” section), *p* < 0.05, false discovery rate marked in bold (n = 4–5).**

### Transcriptomic Analysis Revealed Potential Specialized Metabolite Pathways

**FIGURE 6:**
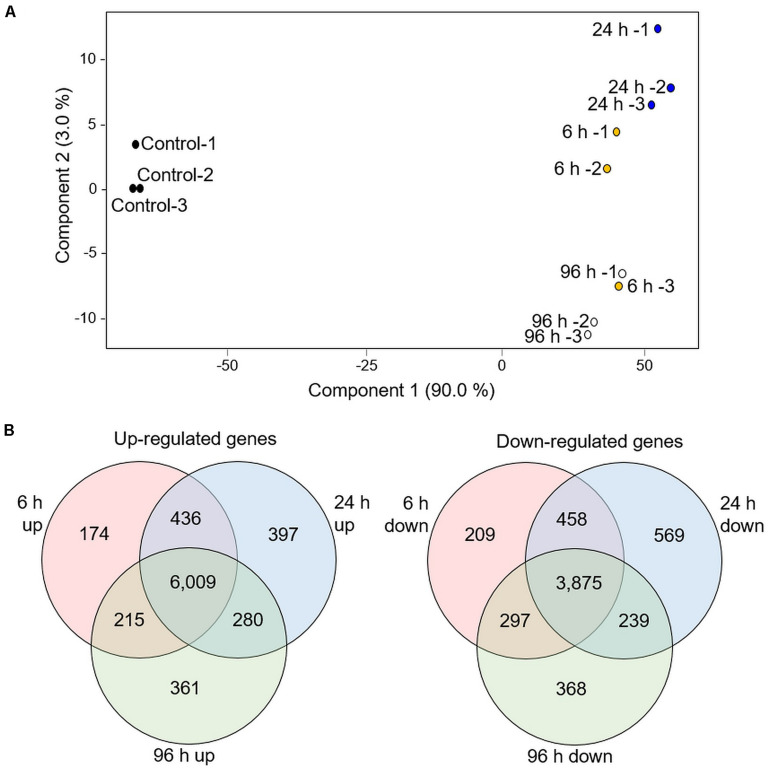
Transcriptomic overview of RTC-144 tef leaves infested with *R. padi* aphid for different periods. **(A)** PCA plot was generated using 35,284 genes. **(B)** Venn diagram illustrating the number of genes up- or down-regulated by aphid infestation in the time course. *p* < 0.05 FDR, and absolute fold change > 2 (*n* = 3 biological replicates for each time point).

We searched for the presence of known deterrent molecules, that were previously reported in other Poaceae family species by comparing the GC-MS data to gramine, and serotonin authentic standards, and HPLC to the benzoxazinoids authentic standards (data not shown). None of these indole-derived compounds were detected. Therefore, we performed a transcriptomic analysis and looked for potential specialized metabolite pathways that are modified in response to aphid infestation. The RTC-144 accession was selected due to its massive variation in VOC metabolism that might relate to the production of other non-volatile specialized metabolite pathways (War et al., 2012). A time-course experiment exposing 1-month-old leaves to *R. padi* for 6, 24, and 96 h, was conducted, and the transcripts were annotated to the gene models found in the *Eragrostis tef* v1.0 reference genome sequence (Cannarozzi et al., 2014). This analysis revealed a total of 35,284 transcripts (Supplementary Table S6). For an overview of the transcriptomic dataset, a PCA plot was constructed on the total tef transcripts. As presented in Figure 6A, the PCA plot indicated that samples from each infestation point were clustered together, with component 1 (90% variance) showing a separation of control and treated samples. Component 2 (3% variance) showed discrimination between 24 and 96 h, while the 6 h samples were divided between these two.

We selected genes with significant expression differences (*p* < 0.05, FDR), and at least a twofold change relative to control, for at least one of the time points (Supplementary Table S7). The total number of up-regulated genes was 7,872, and the down-regulated genes was 6,015 (at least in one of the infestation time points). The distribution of up- and down-regulated genes was calculated for each time point and is presented in a Venn diagram (Figure 6B). Although a unique set of genes was modified at each time point, an impressively large number of genes were detected in the overlap between the three time points (6,009 up-regulated and 3,875 down-regulated genes) These set of genes were associated with defense strategies and metabolic adjustments.

To characterize the metabolic changes occurring in response to aphid attack, an over-representation pathway enrichment analysis was performed on the gene list from each Venn diagram group using the MetGenMAP tool (Joung et al., 2009), comparing the rice orthologs (LOC gene ID; Supplementary Table S8). The super-class of each pathway was categorized by RiceCyc output^5^. Table 4 presents the significantly enriched pathways of up- and down-regulated genes divided into 14 groups. The up-regulated enriched pathways belong to the biosynthesis of specialized metabolites from flavonoids, canavanine, and terpenes. Jasmonic acid biosynthesis, which is a defense-related phytohormone, was enriched upon 24 and 96 h of aphid feeding. In addition, the following pathways were overrepresented: amino acid metabolism (Gly, Cys, Pro, Trp, Asn, Asp, and Arg), nucleoside and nucleotide biosynthesis (purine and pyrimidine), cofactors, prosthetic groups, electron carrier biosynthesis (chlorophyllide a, glutathione), carbohydrate biosynthesis (UDP-D-xylose and dTDP-L-rhamnose) and cell structure biosynthesis (cellulose) (Table 4A). The down-regulated enriched pathways mainly included biosynthesis of specialized metabolites and phytohormones, such as phenylpropanoid biosynthesis, gibberellin, jasmonic acid, cytokinin, and ethylene. Genes from the following pathways were downregulated: carbohydrate biosynthesis (gluconeogenesis and trehalose), carbohydrate degradation (sucrose, starch, and mannose), as well as UDP-glucose conversion and generation of precursor metabolites and energy such as the Calvin cycle, glycolysis and photorespiration were over-represented pathways. Additionally, FA and lipid biosynthesis (acyl-CoA thioesterase and glycolipid), nitrogen metabolism, and Met, Cys, and His amino acid biosynthesis were downregulated (Table 4B). Overall, this suggested massive transcriptomic changes occurring in response to *R. padi* feeding on tef leaves and indicated few potential specialized metabolite pathways that might be involved in tef chemical defense mechanisms.

**TABLE 4:** Enrichment analysis of metabolic pathways.

Treatment (h)	Number of rice homolog genes	Super-class	Pathway	*P*-value	Number of genes
**(A) Up-regulated genes**					
6 up	153	Amino acids degradation	Glycine cleavage complex	2.37E-03	2
		Cofactors, prosthetic groups, electron carriers biosynthesis	Chlorophyllide a biosynthesis	1.96E-02	2
24 up	349	Amino acids biosynthesis	Cysteine biosynthesis	5.19E-04	4
		Cofactors, prosthetic groups, electron carriers biosynthesis	Branched-chain α-keto acid dehydrogenase complex	7.37E-03	3
96 up	323	Carbohydrates biosynthesis/Cell structures biosynthesis	Cellulose biosynthesis	2.83E-02	4
			UDP-D-xylose biosynthesis	9.98E-03	2
			dTDP-L-rhamnose biosynthesis I	2.40E-02	3
		Nucleosides and nucleotides biosynthesis	Purine nucleotides *de novo* biosynthesis	1.86E-02	4
6 and 24 up	385	Cofactors, prosthetic groups, electron carriers biosynthesis	γ-glutamyl cycle (glutathione)	6.49E-03	4
		Nucleosides and nucleotides biosynthesis	Purine nucleotides *de novo* biosynthesis	2.69E-02	4
		Secondary metabolites biosynthesis	Flavonoid biosynthesis	4.23E-02	2
6 and 96 up	190	Carbohydrates biosynthesis/Cell structures biosynthesis	Cellulose biosynthesis	2.24E-03	4
		Secondary metabolites biosynthesis	Flavonoid biosynthesis	8.87E-03	2
24 and 96	253	Carbohydrates biosynthesis/Cell structures biosynthesis	Cellulose biosynthesis	4.00E-03	5
		Nucleosides and nucleotides biosynthesis	Purine nucleotides *de novo* biosynthesis	1.41E-02	4
		Secondary metabolites biosynthesis/Hormones biosynthesis	Jasmonic acid biosynthesis	2.90E-02	3
			Divinyl ether biosynthesis II (13-LOX)	1.56E-03	3
		Secondary metabolites biosynthesis	Mevalonate pathway (terpenes)	4.02E-02	2
All up	3,785	Amines and polyamines degradation	Carnitine degradation	3.99E-02	3
		Amino acids biosynthesis	Arginine biosynthesis II (acetyl cycle)	1.51E-03	9
			Homocysteine biosynthesis	3.03E-02	2
			Asparagine biosynthesis	3.54E-02	4
			Proline biosynthesis (from arginine)	3.99E-02	3
			Proline biosynthesis (from glutamate)	4.54E-02	6
		Amino acids degradation	Tryptophan degradation (side chain pathway)	3.99E-02	3
		Carboxylates degradation/Secondary metabolites degradation	β-D-glucuronide degradation	1.83E-02	3
		Cell structures biosynthesis	Peptidoglycan biosynthesis I	3.99E-02	3
		Carbohydrates biosynthesis/Cell structures biosynthesis	Chlorophyllide a biosynthesis	1.54E-02	11
			Phylloquinone biosynthesis	3.03E-02	2
			Pantothenate biosynthesis (coenzyme A)	4.28E-02	5
		Generation of precursor metabolites and energy	Pentose phosphate pathway (non-oxidative branch)	2.90E-02	5
		Inorganic nutrients metabolism	Urea cycle	1.02E-02	4
		Nucleosides and nucleotides biosynthesis	Purine nucleotides *de novo* biosynthesis I	1.52E-03	20
			*De novo* biosynthesis of pyrimidine deoxyribonucleotides	1.97E-02	8
		Secondary metabolites biosynthesis	Canavanine biosynthesis	3.93E-03	4
**(B) Down-regulated genes**					
6 down	171	Secondary metabolites biosynthesis	Phenylpropanoid biosynthesis, initial reactions	1.56E-03	2
		Secondary metabolites biosynthesis/Cell structures biosynthesis	Suberin biosynthesis	5.06E-03	2
24 down	475	Carbohydrates Biosynthesis	Gluconeogenesis	3.57E-04	8
		Generation of precursor metabolites and energy	Calvin cycle	5.54E-03	6
			Glycolysis	4.07E-02	6
		Cofactors, prosthetic groups, electron carriers biosynthesis	Pyridoxal 5’-phosphate biosynthesis	7.14E-03	2
		Nucleosides and nucleotides biosynthesis	Salvage pathways of purine and pyrimidine nucleotides	4.77E-02	4
96 down	307	Secondary metabolites biosynthesis/Hormones biosynthesis	Divinyl ether biosynthesis II (13-LOX)	9.26E-03	2
6 and 24 down	394	Carbohydrates biosynthesis	Gluconeogenesis	7.09E-03	5
		Carbohydrates biosynthesis/Carbohydrates degradation	UDP-glucose conversion	2.48E-02	4
		Carbohydrates degradation	sucrose degradation to ethanol and lactate (anaerobic)	9.26E-03	8
		Fatty acid and lipid biosynthesis	Acyl-CoA thioesterase pathway	2.15E-02	2
		Generation of precursor metabolites and energy	Glycolysis	7.02E-03	6
6 and 96 down	253	Hormones biosynthesis	Cytokinins-O-glucoside biosynthesis	3.74E-02	5
		Secondary metabolites biosynthesis/Hormones biosynthesis	Divinyl ether biosynthesis II (13-LOX)	1.26E-02	2
24 and 96 down	215	Amino Acids biosynthesis	Histidine biosynthesis	1.13E-02	2
			Methionine salvage pathway	2.20E-02	2
		Hormones biosynthesis	Ethylene biosynthesis from methionine	1.96E-02	2
All down	2,528	Amino acids biosynthesis	Cysteine biosynthesis	3.28E-02	6
			Methionine salvage pathway	1.18E-02	9
		Amines and Polyamines Biosynthesis	Spermine biosynthesis	3.67E-02	3
		Carbohydrates Biosynthesis	Trehalose biosynthesis	3.60E-03	7
		Carbohydrates biosynthesis/Carbohydrates degradation	UDP-glucose conversion	3.43E-02	14
		Carbohydrates biosynthesis/Cell structures biosynthesis	GDP-D-rhamnose biosynthesis	3.96E-02	4
			dTDP-L-rhamnose biosynthesis	4.65E-02	7
		Carbohydrates degradation	Mannose degradation	2.29E-02	3
			Starch degradation	1.66E-02	12
			Sucrose degradation	7.65E-03	5
		Cofactors, prosthetic groups, electron carriers biosynthesis	Ascorbate biosynthesis	2.87E-02	5
		Fatty acid and lipid biosynthesis	Glycolipid biosynthesis	8.95E-06	8
		Generation of precursor metabolites and energy	Photorespiration	2.32E-02	7
		Hormones biosynthesis	Cytokinins-O-glucoside biosynthesis	4.34E-02	26
			Ethylene biosynthesis from methionine	7.75E-03	4
		Inorganic Nutrients Metabolism	Ammonia assimilation cycle II	3.67E-02	3
			Nitrate reduction II (assimilatory)	8.13E-03	9
		Secondary metabolites biosynthesis/Hormones biosynthesis	Gibberellin biosynthesis III (early C-13 hydroxylation)	8.13E-03	4

**Gene expression patterns were sorted into up- and down-regulated genes upon 6, 24, and 96 h of aphid feeding on RTC-144 tef plants.**

## Discussion

### Tef Plants Grown in a Mediterranean Climate Are Hosts for Insects From Seven Different Orders

Our study is the first report of insect groups associated with tef crops grown in a Mediterranean climate. Seven orders were detected in our survey: Coleoptera, Diptera, Hemiptera, Hymenoptera, Lepidoptera, Neuroptera, and Orthoptera (Figure 1). In Ethiopia, the major insect pests of tef plants are the Wello-bush cricket (*Decticoides brevipennis*, order Orthoptera), the barley fly (*Delia arambourgi*; order Diptera), the black tef beetle (*Erlangerius niger* Weise; order Coleoptera), the Mendi termite (*Macrotermes subhyalinus*; order Isoptera), and red tef worm (*Mentaxya ignicollis*; order Lepidoptera). Among the minor pest abundance in Ethiopia are two aphids species from the Hemiptera order, Russian wheat aphid (*Diuraphis noxia)* and corn leaf aphid (*Rhopalosiphum maidis*), and desert locust (*Schistocerca gregaria*; order Orthoptera) (Gebremedhin, 1987; Stallknecht et al., 1993), and Insect Pests of Cereals in Ethiopia database^6^. There are some similarities between the insect orders in Israel and Ethiopia, but not in the insect abundance. In Israel, the most abundant insects in Sede Boqer (three tef accessions) and Revadim (two accessions) were Hemipterans and their three families, Pentatomidae, Cicadidae, and Aphidoidea (Figure 1).

In Israel, 194 aphid species were reported, and many of them are fed on Poaceae family plant species (Swirski and Amitai, 1999). Interestingly, green lacewings (*Chrysopa perla*), from the Neuroptera order and Chrysopidae family, were also spotted in the field. Larvae of this species are documented to be voracious predators feeding on aphids and other soft-bodied arthropods, therefore serving as a biocontrol of aphids (Tauber et al., 2000). Increasing vegetation biodiversity in agroecosystems can impact the abundance of insect herbivory and their natural enemies (Knops et al., 1999). Tef is commercially grown in Israel since 2014 at a minor scale and might change the vegetation biodiversity. If tef cultivation expands, it might affect insect pests, depending on the insect’s ability to use a wide range of plants such as wild and cultivated Poaceae plant species as well as alternative hosts.

### Aphid Reproduction, Preference, and Feeding Behavior Are Different Between the Three Selected Accessions

Aphids are major agricultural pests worldwide and are considered a common pest on Poaceae family plant species such as maize, wheat, barley, and millets (Robinson and Hsu, 1963; Kalaisekar et al., 2017). The non-choice bioassay indicated that aphids reproduced in all three accessions; RTC-406 was the most aphid resistant among the three accessions (Figure 2B), while the choice bioassay revealed that RTC-144 is the most repelling (Figure 2A). The EPG results imply that the aphids settled and started probing more swiftly on the leaf of RTC-144 and spend less time non-probing than RTC-406. A recent study assessed the potential surface resistance of sorghum plants to sugarcane aphids (*Melanaphis sacchari*) and suggested that the aphids spend approximately twice longer in the non-probing phase in the resistant plants than in the susceptible plants (Tetreault et al., 2019). Barley leaves infested with *R. padi* showed a shorter time of salivation and ingestion of the phloem on resistant relative to the susceptible plants. Feeding patterns reflect many factors, including mechanical barriers present at the leaf surface, olfactory repellents, and host metabolism (Tetreault et al., 2019). The results highlight the need for conducting multiple bioassays combined with metabolic and transcriptomic methods to expose the mode of defense.

### Non-granular Trichome Density Is Negatively Correlated With Aphid Reproduction and Might Affect Feeding Behavior

Tef leaves are covered with non-glandular trichomes (epidermal hair-like structures). Similar structures were observed on wheat and barley leaves (Leybourne et al., 2019; Correa et al., 2020). The non-glandular trichomes serve as a physical barrier that can limit insect movement and interrupt the stylet insertion of phloem feeders (Handley et al., 2005; Sato and Kudoh, 2015). Trichome density can vary by leaf position, development stages, genetic backgrounds, and even be induced upon insect attack (Leybourne et al., 2019). The RTC-406 accession possessed the highest trichome levels in all three leaves, suggesting the combined impacts of leaf position and genotype (Figure 3). Trichome density was negatively correlated with aphid reproduction (Batyrshina et al., 2020b), suggesting the role of non-glandular trichomes on tef leaves as a partial defense strategy. The trichome destiny and feeding behavior results emphasize that the high number of trichomes of RTC-406 tef leaves, is one of the factors that might extend the time of aphid penetration to the tef leaf tissue. The time spent by aphids in the phloem stage is linked primarily to feeding as well as acquisition and transmission of viruses and bacteria (Martin et al., 1997). However, we found no significant difference between RTC-406 and RTC-144 in the phloem phase. Aphids are phloem feeders that occasionally feed on xylem fluid (Nalam et al., 2020), possibly to attenuate the high osmotic potential of the phloem sap (Douglas, 2006; Tetreault et al., 2019). The EPG results expose that aphids spent more time ingesting sap in the xylem on RTC-144 than RTC-406. This might be due to differences in the constitutive levels of glucose between the two accessions (Figure 5), which is known to determine the osmotic potential of the phloem sap (van Bel and Hess, 2008). The results suggest that the factors involved in tef resistance are found not only on the surface but also in phloem and xylem composition.

### Tef Plants Synthesize a Rich Blend of Volatile Compounds

Tef plants synthesized VOCs from five different metabolic classes (Supplementary Table S2). A recent study on two grasses, itchgrass (*Rottboellia cochinchinensis*) and African star grass (*Cynodon nlemfuensis*), showed that their VOC profile is composed of metabolites from different classes (Ramírez-Medorio et al., 2019). In contrast, wheat and maize main VOC classes are terpenoids and FA derivatives (including the GLV), which are associated with defenses (Richter et al., 2015, 2016). Although several mono-, and sesqui-terpenes have shown repellent properties to insects (Bleeker et al., 2009), none of the compounds from the terpene class were significantly modified in tef (Table 4), which suggests that other VOC classes might play a defensive role. Furans were only detected in fleshy fruits during ripening stages (Klein et al., 2007), but were not previously reported in vegetative tissues. This class might be unique for tef volatiles and might demonstrate that VOC compositions are species-specific (Nordlund et al., 1977).

### Aphid Host Preference Mediated by Volatiles in Tef Plants

Volatile compounds have broad ecological functions as olfactory repellents or attractants (Bernasconi et al., 1998; Jimenez-Martínez et al., 2004; Piesik et al., 2008). For example, (E)-2-pentenal (GLV class), and FA esters are known to have anti-feedant properties to aphids (Hammond et al., 2000; Santana et al., 2012). The VOC profile revealed that untreated RTC-144 plants, produced high levels of GLV, furans, and irregular terpenes and low levels of ester FA derivatives compared to RTC-406 (Figure 4). This accession repelled the *R. padi* aphids in the olfactometer choice bioassay (Figure 2A), which emphasizes that the VOC composition of RTC-144 has constitutive repellent properties. In response to aphid infestation, ester FA derivative levels increased while some of the aliphatics were reduced in RTC-144 (Figure 3). These results suggest that FA derivatives might have a potential function as attractants of predators and parasitoids (Kessler and Baldwin, 2002; Shiojiri et al., 2006); this requires further investigation. Methyl hexanoate was significantly increased in all three accessions (Table 2). This compound was previously reported to act as insect attractant pheromone of Mediterranean fruit fly (*Ceratitis capitate*) in peach plants (*Prunus persicae*), and found in low levels in the least susceptible cultivars (Tabilio et al., 2013). The ecological function of methyl hexanoate produced by the tef plants is yet unknown.

### Constitutive and Inducible Alternation of Central Metabolism Profiles Upon Aphid Infestation

Numerous changes in the central metabolism of plants occur in response to insect herbivory, including the alternation of photosynthetic efficiency, remobilization of carbon and nitrogen resources, and regulation of plant growth rate (Zhou et al., 2015). The metabolic analysis of tef leaves infested with *R. padi*, indicated a shift in the biosynthesis of carbon-rich compounds (sugars and organic acids), toward nitrogen-containing compounds (amino acids). Modification of the amino acid composition and levels can reduce plant palatability and nutritional quality in the phloem sap, and contribute to increased resistance to aphids (Karley et al., 2002). The infestation of *R. padi* on barley plants under nitrogen-deficient growth conditions exhibited reduced reproduction rates relative to aphids exposed to plants grown under nitrogen-rich conditions (Ponder et al., 2000, 2001). This can be determined by the composition of amino acids. Previous studies reported that upon aphid infestation, the levels of essential amino acids were elevated in susceptible plants (Vogel and Moran, 2011; Leybourne et al., 2019). Similarly in the three tef accessions, both essential amino acids (Val and Leu), as well as a non-essential amino acid (Gly), were increased upon aphid attack (Table 3 and Figure 5). Cereal aphid species actively remobilize wheat and barley nutrients in the phloem to increase the abundance of amino acids, while *R. padi* seems to have a slight effect on amino acid composition (Sandström et al., 2000; Leybourne et al., 2019). To better understand the metabolic changes in the tef leaves, further metabolic analysis of the phloem sap is required.

### Transcriptional Changes of Infested RTC-144 Points to Metabolic Pathways That Might Be Involved in Chemical Defenses

The tef transcriptome was dramatically modified in response to aphid infestation. The effect of insect feeding on plant leaves is a dynamic process that continually changes according to exposure time (Tzin et al., 2015). A recent time-course transcriptomic analysis of wheat leaves infested with *S. graminum* aphids reported that approximately 10,000 genes were significantly altered (Zhang et al., 2020). In the tef leaves, the expression levels of 13,887 genes were significantly altered within 6 h and continued to change during the entire 96 h experiment (Figure 6). Herbivory causes changes in the expression of genes involved in both central and specialized metabolism (Appel et al., 2014). In the tef transcriptome analysis, the up-regulated enriched pathways included amino acid metabolism, and biosynthesis of nucleosides and nucleotides, cofactors, prosthetic groups, electron carriers, carbohydrates, and cell structures. The downregulated enriched pathways mainly included FAs and lipids, inorganic nitrogen metabolism, and amino acid biosynthesis (Met, Cys, and His), carbohydrate biosynthesis, carbohydrate degradation, glucose conversion and generation of precursor metabolites and energy such as through the Calvin cycle, glycolysis, and photorespiration. The observed reduction in carbohydrate metabolism and generation of precursor metabolites and energy pathways combined with modification in the biosynthesis of phytohormones such as jasmonic acid has been reported as the result of regulation of resource-based trade-offs between growth and defense (Mitra and Baldwin, 2014).

The transcriptomic dataset indicated that the gene expressions of different classes of specialized metabolites were over-represented, including flavonoids, canavanine, and terpenes. The accumulation of flavonoids, including the subclass flavones, flavonols, and anthocyanins, was enhanced in pea seedlings (*Pisum sativum*) in response to attacks by the pea aphid (*Acyrthosiphon pisum*) (Morkunas et al., 2016). Canavanine is a non-protein toxic amino acid, structurally related to the amino acid Arg. It is highly abundant in seeds and sprouts of many legumes and possesses insecticidal properties to most insects (Rosenthal, 2001; Mitri et al., 2009; Staszek et al., 2017). Terpenes have defensive properties such as volatile metabolites or non-volatiles such as triterpene saponins (Fürstenberg-Hägg et al., 2013; Liu et al., 2019). These three pathways should be further investigated as potential defensive compounds in tef.

## Conclusion

While the world depends on many crop species, the commercialization of conventional agriculture is limited to a few cereal crops, mainly wheat, rice, and maize. Traditional crops, such as tef, are important resources for improving agricultural diversity, production, nutritional qualities, and increasing food security (Padulosi et al., 2012). Therefore, further investigation is required to understand understudied crop plants such as tef and other millets. In this research, we explored the molecular mechanisms involved in the interaction between *R. padi* and tef by comparing them to the well-studied physical and chemical mechanisms used by other crops such as wheat and barley. We discovered that tef plants use similar defense mechanisms; however, the indole-derived toxic compounds present in these crops were not synthesized by tef leaves. Here, we suggest three potential specialized metabolite pathways that might function as deterrent metabolites, which requires further investigation. Notably, in this research, only three accessions were tested that represent a random sampling of the variation in tef and were not selected based on aphid resistance. The tef germplasm might exhibit stronger resistant and susceptible accessions than the ones that we tested. To fully understand how well tef adapted to aphids, there is a need to conduct a large-scale experiment and exploit the most resistant accessions to better understand deterrent molecules involved in defenses. The overall understanding of tef biotic challenges and their responses are essential for the development of strategies to control pest infestations and reduce yield loss in worldwide cereal crops, supporting global food security.

## Data Availability Statement

The datasets presented in this study can be found in online repositories. The names of the repository/repositories and accession number(s) can be found in the article/Supplementary Material.

## Author Contributions

NG, BY, YS, and VT conceived and designed the experiments. NG, NW, AS, AC, and SB-Z performed the experiments. NG, BY, NW, AC, and VT analyzed the data. NG, BY, SB-Z, YS, and VT contributed to the writing of the manuscript. All authors contributed to the article and approved the submitted version.

## Conflict of Interest

The authors declare that the research was conducted in the absence of any commercial or financial relationships that could be construed as a potential conflict of interest.

## Footnotes

1 https://imagej.nih.gov/ij/

2 http://gmd.mpimp-golm.mpg.de/

3 www.genewiz.com

4 www.jmp.com

5 http://pathway.gramene.org/

6 https://ethiopia.ipm-info.org/

## Supplementary Material

Supplementary Figure 1 A photo of the three selected tef accessions. The plants are 1-month-old.

Supplementary Figure 2 Photos of several insects identified in the field survey.

Supplementary Figure 3 The Y-shape tube olfactometer system used for this experiment.

Supplementary Table 1 Total insect identified in the two field sites, Sede Boqer and Revadim, at two sampling dates in May and June 2019. The insects were divided into orders and families.

Supplementary Table 2 Known VOCs identified in leaves of 1-day-old tef plants infested with aphids for 4 days. The metabolites were normalized to the internal standard and presented as the relative abundance of the ion counts.

Supplementary Table 3 VOCs that were significantly different between tef accession, treatment (with and without aphid infestation), or through the interaction of these factors using two-way ANOVA.

Supplementary Table 4 Central metabolites identified in leaves of 1-day-old tef plants infested with aphids for 4 days. The metabolites were normalized using GC-MS to the internal standard and presented as the relative abundance of the ion counts.

Supplementary Table 5 Central metabolites that were significantly different between tef accession, treatment (with and without aphid infestation), or through the interaction of these factors using two-way ANOVA.

Supplementary Table 6 Total RNA-seq values after rlog normalization. Gene IDs were determined by *Eragrostis tef* reference transcriptome version 1.0.

Supplementary Table 7 Distribution of RTC-144 tef genes into the seven Venn diagram groups. The genes were either up-, or down-regulated by aphid infestation in the time course. *p*< 0.05 false discovery rate, and fold change > 2 or < 0.5.

Supplementary Table 8 Distribution of rice orthologs of tef genes, divided into the seven Ven diagram groups. The genes were either up-, or down-regulated by aphid infestation in the time course. *p* < 0.05 false discovery rate, and fold change > 2 or < 0.5.
