# Supplementary material for: Variation Between Three Eragrostis tef Accessions in Defense Responses to Rhopalosiphum padi Aphid Infestation
Source: Front Plant Sci. 2020 Dec 8;11:598483. doi: 10.3389/fpls.2020.598483 (PMC7752923; doi:10.3389/fpls.2020.598483)
Supplement: Supplementary Figure 1 — A photo of the three selected tef accessions. The plants are 1-month-old. [file Data_Sheet_1.PDF]

Figure S1

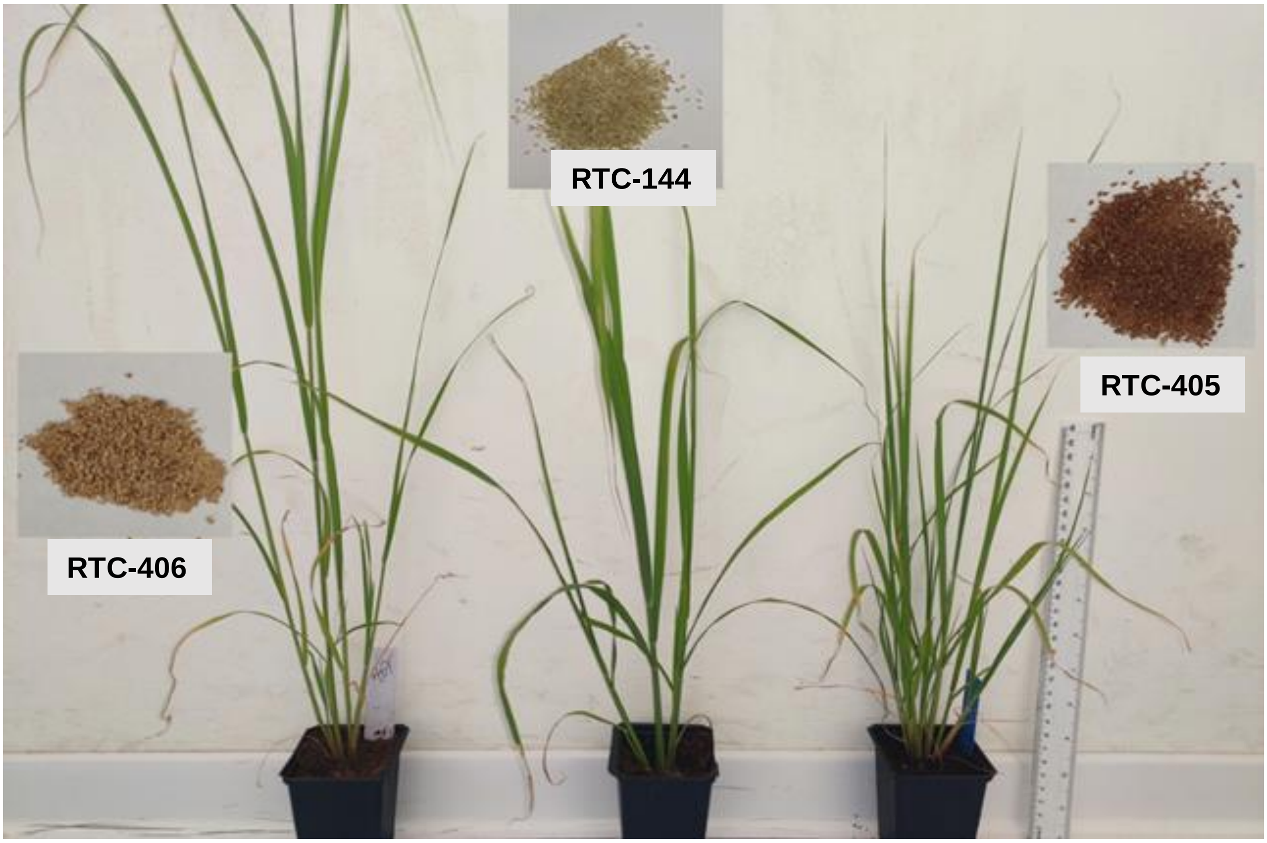

Figure S1. A photo of the three selected tef accessions. The plants are one-month old.

Figure S2

Forest shield bug (*Oncacontias vittatus*)

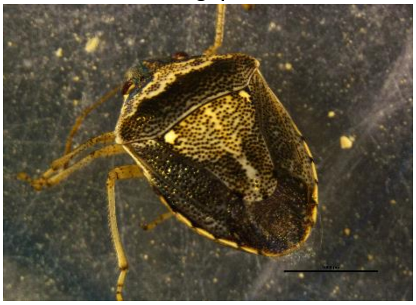

Green shield bug (*Palomena prasina*)

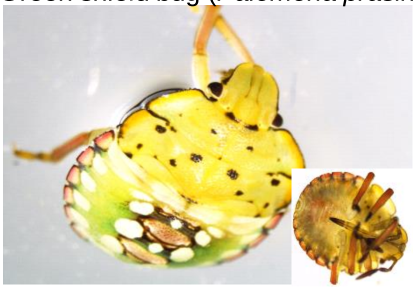

Bird cherry-oat aphid (*Rhopalosiphum padi)*

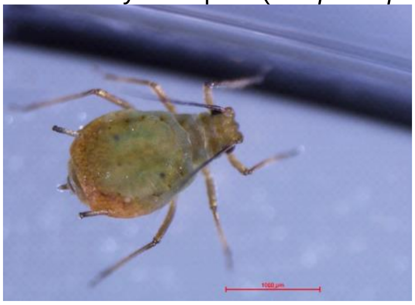

Green lacewings (*Chrysopa perla*)

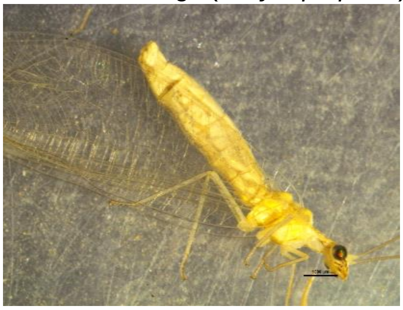

Figure S2: Photos of several insects identified in the field survey.

Figure S3

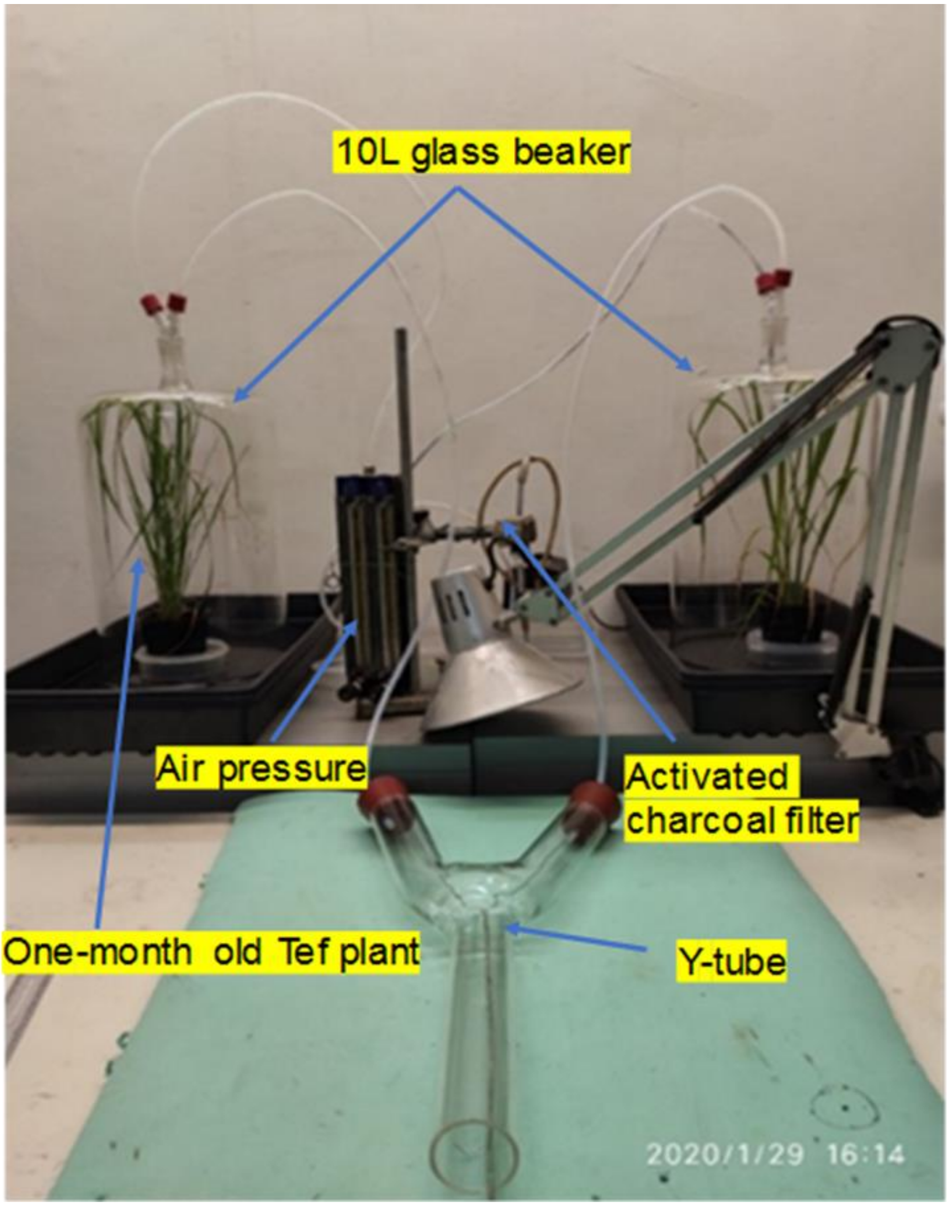

Figure S3. The Y-shape tube olfactometer system used for this experiment.
